# Magnetic Field Triggered Multicycle Damage Sensing and Self Healing

**DOI:** 10.1038/srep13773

**Published:** 2015-09-08

**Authors:** Anansa S. Ahmed, R. V. Ramanujan

**Affiliations:** 1 School of Materials Science and Engineering, Nanyang Technological University, 639798, Singapore

## Abstract

Multifunctional materials inspired by biological structures have attracted great interest, e.g. for wearable/ flexible “skin” and smart coatings. A current challenge in this area is to develop an artificial material which mimics biological skin by simultaneously displaying color change on damage as well as self healing of the damaged region. Here we report, for the first time, the development of a damage sensing and self healing magnet-polymer composite (Magpol), which actively responds to an external magnetic field. We incorporated reversible sensing using mechanochromic molecules in a shape memory thermoplastic matrix. Exposure to an alternating magnetic field (AMF) triggers shape recovery and facilitates damage repair. Magpol exhibited a linear strain response upto 150% strain and complete recovery after healing. We have demonstrated the use of this concept in a reusable biomedical device i.e., coated guidewires. Our findings offer a new synergistic method to bestow multifunctionality for applications ranging from medical device coatings to adaptive wing structures.

Multifunctional materials have attracted intense interest for the development of next generation technologies^1^,^2^. Often drawing inspiration from nature, such multifunctional composites exhibit a range of properties and designs^3^. Building blocks can be combined to produce advanced materials, similar to the biological approach, e.g., of combining amino acids into complex proteins. Thus, the ultimate goal is to create higher forms of materials which can mimic “life” functions^4^. The life functions of damage sensing and healing can increase reliability and service life^5^,^6^; triggering these functions remotely and wirelessly is advantageous in industrial and engineering components and coatings, especially in components which are not easily accessible or must remain *in situ*.

Specifically, there is a significant need for sensing and healing in polymeric materials which undergo mechanical stresses during service, resulting in severe plastic deformation and failure. For example, polymer composite skins in morphing aircraft wings and biomedical coatings used in catheters and guidewires during minimall y invasive surgery can benefit from damage sensing and healing^7^,^8^,^9^. Half the mass and cost of a transport aircraft wing is due to the complex high-lift system, which could potentially be replaced by lighter and simpler morphing systems^10^. Morphing wing aircraft have lower engine thrust requirements and ~8% lower take-off gross weight compared to fixed-geometry wing aircraft^11^. The development of such morphing wings requires durable outer skins, which can benefit from self healing and damage detection^8^,^12^,^13^.

For biomedical catheter and guide wire coatings the U.S. Environmental Protection Agency requires that manufacturers eliminate the suspected carcinogen perfluorooctanoic acid (PFOA) from their PTFE formulations by 2015. This is a challenge due to flaking and delamination of PTFE coatings, which has resulted in FDA recalls of several types of guidewires produced by various companies^13^. Thus, there is significant demand for a new reusable coating capable of damage detection and healing before each use.

Materials exhibiting damage sensing and self healing, also referred to as “electronic skins”, are often based on conductive materials, which can sense strain and/or damage^14^,^15^,^16^. However such materials cannot be applied to large structures due to the disadvantage that they have to be permanently connected to a power source. For example, in aerospace applications or structural health monitoring materials, it is impractical to permanently connect all parts of the structure to a power source. Visible color change on damage followed by self healing has also been recently reported by Urban *et al.*^17^ Bleeding of fluorescent markers due to damage and self healing *via* infiltration of the damaged region by a healing agent supplied through hollow fibers has been demonstrated^18^.

Chromic materials exhibit color emission or intensity change induced by external stimuli such as temperature, solvent, light, electron radiation, pressure, humidity, etc. Recently, mechanochromic polymers have shown promise to introduce strain, temperature and solvent sensing in polymers^19^,^20^,^21^,^22^,^23^,^24^. Mechanochromic molecules act as strain sensors due to their tendency to aggregate in amorphous regions of a thermoplastic semicrystalline polymer. Upon aggregation, they undergo a change in their emission spectrum, which is detectable in the visible region. When the polymer undergoes plastic deformation, the aggregates break up into monomers with concurrent change in the emission spectrum^25^,^26^,^27^, indicating that damage has occurred and a healing cycle is necessary. A significant improvement to the current level of functionality would be to impart multiple cycle damage sensing to a thermoplastic matrix using mechanochromic molecules and shape memory polymers (SMP)^28^. Combining such multi cycle damage sensing *via* color change with triggered healing results in materials exhibiting life like properties^29^.

For self healing, the wound closure method is particularly attractive for polymers. It is a macroscale healing technique commonly observed in biological systems^30^. As wounds heal there is a change in the mechanical stress as material is redistributed around the damaged region. Shape memory assisted self healing (SMASH) can be used to mimic this biological behaviour^31^,^32^. SMASH occurs when the shape memory transition temperature of the polymer is exceeded and the polymer contracts, restoring the original sample geometry. This can be followed by polymer chain entanglement across the damaged interface (when the damaged sections are in contact) ultimately resulting in healing. The SMASH method has been used to achieve repeated healing in corrosion resistant coatings^33^.

Magnet-polymer composite (Magpol) materials are well suited for damage sensing and self healing in both large structure morphing wing skins and small scale biocompatible guidewire coatings. Prior work has shown that, due to flexibility in the choice of polymer matrix and magnetic fillers, remote contactless actuation, high actuation strain and large strain rate, self-sensing and quick response can be achieved^34^,^35^,^36^. Here we report the development of magnetically responsive Magpol with multicycle strain sensing and locally and remotely triggered healing. Magpol consists of magnetic Mn-Zn ferrite nanoparticle filler in a commercial poly (ethylene-co-vinyl acetate) (EVA) thermoplastic matrix. The magnetic filler can be used to trigger healing by locally heating the composite by employing an external alternating magnetic field (AMF)^37^,^38^,^39^. The thermoplastic matrix ensures multiple strain sensing cycles and self healing through a shape memory mechanism.

The novelty of this work lies in the development of damage sensing and healing functionalities to commercial polymers. The magnetic filler can trigger actuation, self healing and multiple cycle damage sensing. We have, for the first time, imparted both multi cycle damage sensing and self healing to a commercial thermoplastic without chemical modification.

The shape memory of the matrix plays a crucial role in this multifunctionality: (a) Magpol can return to its original shape following an extended period of large inelastic deformation. Application of an external temperature stimulus triggers the shape memory property, resulting in healing. The temperature stimulus is generated *via* an applied alternating magnetic field (AMF), which induces heating of the magnetic nanoparticle filler. This heating is controlled by tuning the Curie temperature of the nanoparticles to correspond to the temperature required for the reaction. Controlled heating of magnetic nanoparticles to trigger reactions has been previously studied by Ahmed *et al.*, Bowman *et al.* and McHenry *et al.*^40^,^41^,^42^,^43^,^44^,^45^ The resulting temperature rise triggers recovery of the original dye aggregates, enabling repeated multi cycle sensing and repair. this shape recovery has been used for healing^46^. (b) Magpol can act as a “memory chromic polymer”; the shape memory effect (SME) can be exploited during inelastic deformation to change color, which can reveal damage and indicate successful repair.

Figure 1 is a schematic of the strain sensing and triggered healing of Magpol. Plastic deformation causes color change from greenish blue to deep blue due to disaggregation of the bis(benzoxazolyl)stilbene (BBS) chromophore. Further deformation ultimately leads to failure. Following failure, the two pieces of Magpol are placed in contact and exposed to an external radio frequency alternating magnetic field (AMF). The magnetic nanoparticles generate heat due to Néel relaxation losses which is transferred to the surrounding shape memory polymer matrix (poly ethylene –co- vinyl acetate EVA). The Mn Zn ferrite nanoparticles used in this study have a Curie temperature of 230°C which is below the temperature at which EVA undergoes pyrolysis, thus ensuring the stability of the system^45^. The resulting temperature rise triggers recovery of the original component shape and the original chromophore aggregate structures. This is followed by polymer chain entanglement at the failure interface of the two pieces; leading to healing. The advantage of using magnetically triggered heating is that heating can be remotely triggered at high penetration depth. Secondly, chromophore aggregates on the surface of Magpol are at relatively lower temperatures due to heat loss to the surroundings, preventing their disaggregation. The interface to be healed can, in the meanwhile, achieve the higher temperature required for polymer chain movement, ensuring efficient healing.

## Results

### Characterization

Magnetic nanoparticles with the composition Mn_0.8_Zn_0.2_Fe_2_O_4_ were synthesized with an average size of 12 nm, as determined from XRD and TEM micrographs. The saturation magnetization of the nanoparticles as measured by VSM was 65 emu/g, the particles showed negligible hysteresis, indicating their superparamagnetic nature.

It was found that the BBS chromophore concentration of 0.1% yielded aggregates which undergo disaggregation under strain. Annealing time of ~80 min. was also found to be sufficient to produce chromophore aggregates.

The Magpol composites were synthesized with 12 wt%, 16 wt% and 20 wt% nanoparticle loading. Less than 10 wt% loadings, did not generate enough heat in the AMF to trigger shape recovery or healing. Loading greater than 20 wt% caused the BBS chromophore to dissolve completely in the matrix in the form of single molecules, changing the emission spectrum.

DMA results (Fig. 2) show that there is not much change in Tg with increased filler loading. The Tg ranges between −25 °C for 0 wt% loading to −20 °C for 20 wt% loading. At −80 °C, there is an increase in the storage modulus with loading. The storage modulus was found to be 1.5 GPa, 1.8 GPa, 2 GPa and 2.6 GPa for 0 wt%, 12 wt%, 16 wt% and 20 wt% loading respectively (supplementary information). The shape memory properties were measured by unconstrained strain recovery. It was observed that, with increased filler loading, increasing stress was required to achieve the same strain, while shape recovery improved with increased loading. Complete shape recovery can be observed when Magpol is heated above 86 °C. However, due to increased sample slippage at the grips at higher temperature, the maximum recovery temperature was fixed at 70 °C. At 70 °C there is a small difference in the shape recovery properties of Magpol; increasing loading enables higher shape recovery, EVA 0 wt% filler shows the least recovery.

### Strain Sensing in Magpol

Photoluminescent (PL) measurements were conducted to investigate the correlation between the shape memory effect and the chromic properties of Magpol. The 20 wt% Magpol was deformed at room temperature until failure (Fig. 3). The emission spectrum in the center of the deformation region was measured for each strain value. Upon plastic deformation the chromophore aggregates (I_E_) (515 nm) exhibited distinct changes in the absorption band compared to the monomeric species (I_M_) (475 nm). There is an almost linear decrease in the I_E_/I_M_ ratio up to 150% strain, followed by a slower decrease until failure.

### Shape memory response

**Figure 1:**
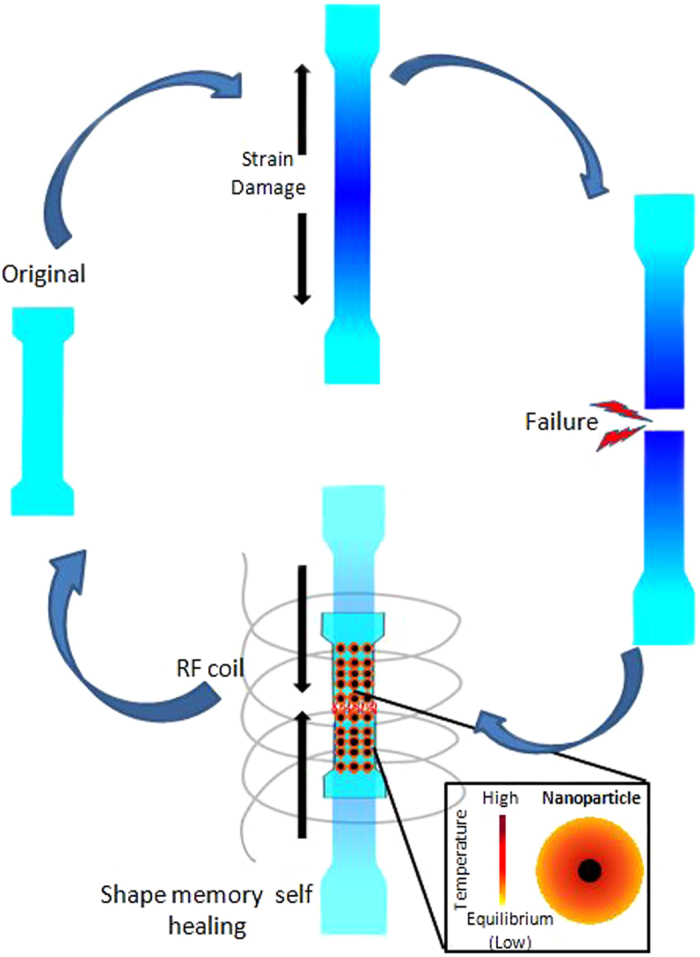
Schematic of damage sensing and self healing in Magpol. The sample displays damage sensing by a colour change on plastic deformation. This colour change becomes more pronounced with increasing strain. The sample is strained until failure. Subsequently, healing is carried out in an AMF. Embedded nanoparticles generate heat, resulting in recovery of the original shape and healing of the damaged region along with a return to the original colour.

Shape recovery of Magpol was carried out by placing samples in an AMF, the magnetic nanoparticle filler provides sufficient heat *via* relaxation losses to trigger the shape memory effect. Table 1 shows the shape fixity and recovery values of 12 wt%, 16 wt% and 20 wt% Magpol over three cycles of strain and recovery. As expected, increased loading generates more heat, resulting in higher shape recovery. The initial cycle had lower shape recovery, subsequent cycles showed better performance. This could be due to alignment of the hard segments in the strain direction during the first cycle. This alignment is retained during subsequent cycles, improving shape recovery. The DMA analysis also supports this result, less stress is required during the second cycle to achieve the same strain. (Fig. 1).

### Multicycle damage sensing

Previous studies have shown the use of mechanochromic molecules as strain sensors in polymers^21^. However, a significant drawback is the single cycle use. In previous reports, plastic deformation could not be reversed hence the technique was only applicable for one time use^26^. Other reports demonstrated repeatable sensing in elastomers through chemical modification of the polymer chains^47^. On the other hand, in our experiments, we use shape memory recovery to reorganize BBS chromophore aggregates as well as to recover from plastic deformation. Magpol, with different filler loading, was subjected to three cycles of plastic deformation (~200%) and recovery in an AMF. The I_E_/I_M_ ratio was recorded before and after strain deformation as well as after recovery. The percent change in the I_E_/I_M_ ratio was calculated for each case and recovery was determined (Fig. 4). It was observed that changes in emission spectra upon damage and recovery increased with filler loading.

The photoluminescent intensity of Magpol does not decrease over consecutive cycles, indicating that heat generated by the magnetic nanoparticle filler does not degrade chromophore molecules. Additionally, atomic force microscopy (AFM) images also show recovery of the initial surface roughness of the film, indicating that chromophores aggregated after shape recovery (Supplementary information).

### Self Healing

The shape recovery of the plastically deformed region described above can be used to develop a self healing system and repair Magpol that has undergone failure.

Healing of Magpol occurs in two steps, (a) shape recovery which restores the original sample dimensions. This step reverses the plastic deformation that has occurred before failure, resulting in two pieces with a defined edge (Fig. 5(A ii)). During this process, the original chromophore aggregates are reformed, resulting in the recovery of the original colour (Fig. 5(B)). The fluorescence intensity of the chromophore aggregates in the recovered sample is not equal to the original, but substantial restoration is still observed which is sufficient to cause a colour change. (b) the two halves are then held together in an AMF for 10 min. to achieve complete healing at the interface (Fig. 5(A iii)). Healing is achieved through entanglement of polymer chains along the interface. Figure 5 shows the recovery of mechanical properties of Magpol after failure. The yield stress is lower in the recovered sample, possibly due to lower crystalline content (consistent with the DSC and XRD analysis: Supplementary information). Crystallites can act as fillers and as cross-links which are not completely recovered after failure. Multiple cycles of self healing have been studied in our previous work^45^.

A practical application of such coatings is in reusable biomedical devices. As a proof-of-concept of self healing, we coated a NiTiNOL guidewire of diameter 0.8mm with Magpol. The coating was achieved through dip-coating a 20 wt% nanoparticle solution of EVA onto the NiTiNOL guidewire to achieve an outer diameter of 1.2mm. Wear damage was simulated by pulling the guidewire through a plastic ring of slightly larger diameter, as would occur in a practical setting during insertion and removal of the guidewire from the body. The damaged section of the wire was then healed in an AMF for 20 min. First, the protruding portions of the damaged area (indicating plastic deformation) returned to their original shape. With further exposure, the original smooth surface was recovered, resulting in healing (Fig. 6).

## Discussion

MnZn ferrite nanoparticles were chosen as the filler because their Curie temperature (230 °C) is less than the degradation temperature of the matrix (>300 °C) and the melting point of the BBS chromophore (360 °C), which prevents accidental overheating of the sample in the magnetic field. EVA was used as the matrix because of its shape memory properties and its semicrystalline nature.

**Figure 2:**
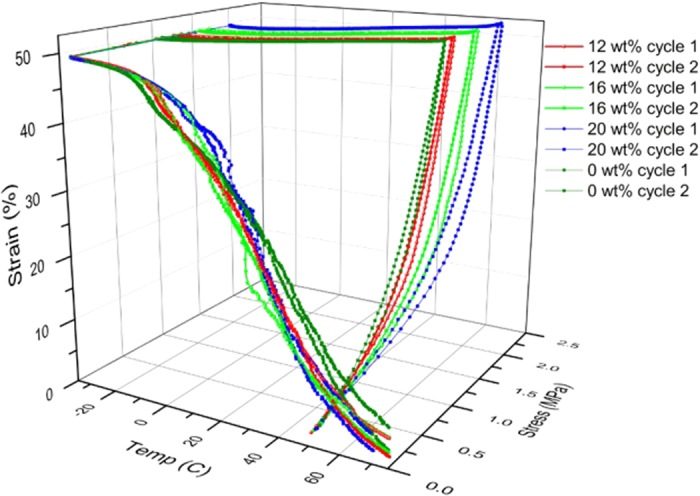
DMA measurement of the Shape memory properties of Magpol with filler concentrations of 0, 12, 16, and 20 wt% over two cycles of unconstrained strain and recovery.

**Figure 3:**
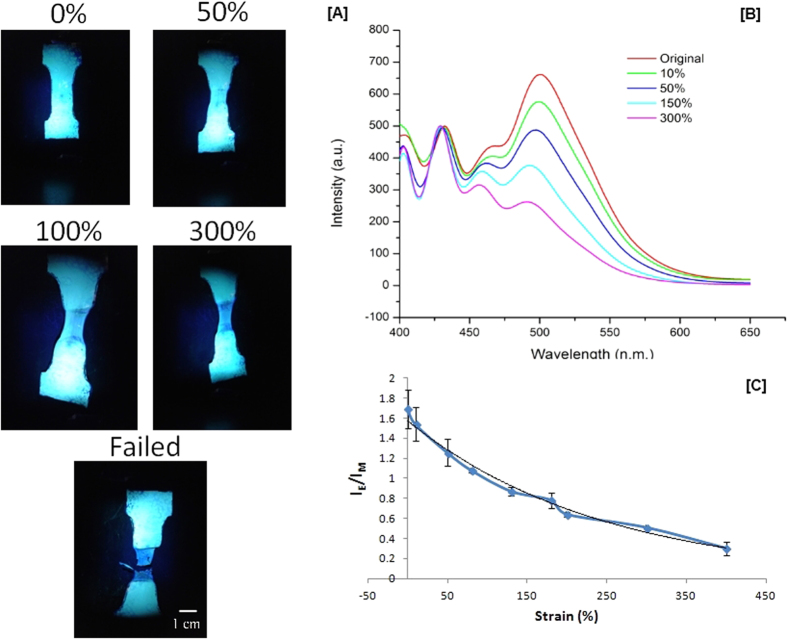
Strain sensing in Magpol. (**A**) Color change due to plastic deformation observed under UV light @ 377 nm. Deformed regions are deep blue , undeformed material is bluish green. (**B**) Photoluminescent spectra of Magpol shows the change in the relative heights of the peaks at 425 and 515 nm, which indicate the original colour and colour after straining respectively; The peak at 515 nm becoming more pronounced with increasing strain. (**C**) Sensitivity of Magpol to a range of strain values measured by the relative change in intensity of 425 and 515 nm peaks as a function of % strain.

**Figure 4:**
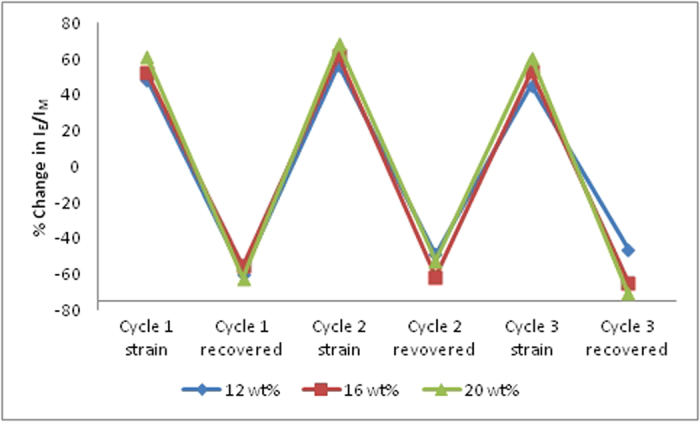
Change in relative intensities in the photolumenscent spectra of Magpol at 425 and 515 nm (plotted as a ratio I_E_/I_M_ following strain and shape recovery over 3 cycles). Cycling was done for 12, 16 and 20 wt% filler samples.

**Figure 5:**
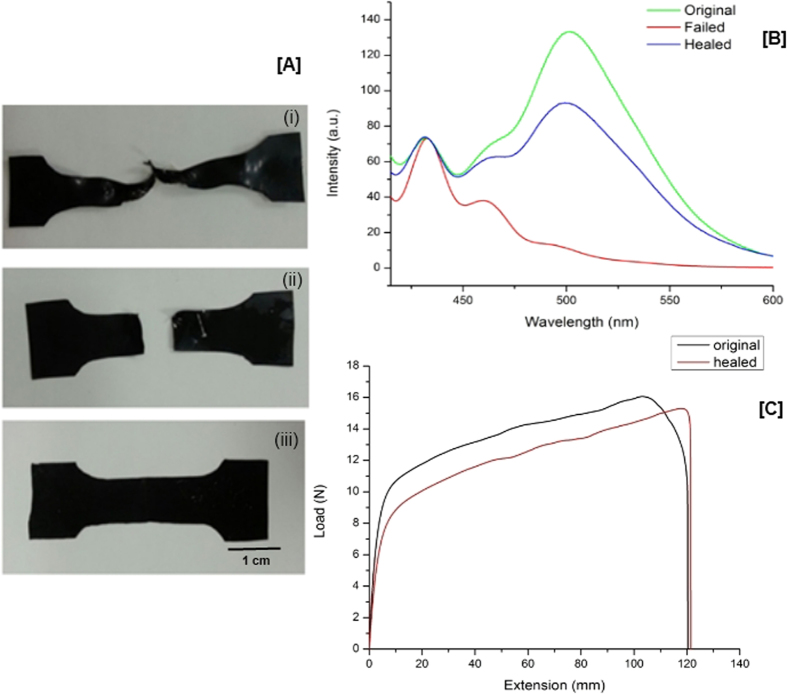
Self healing properties of Magpol. (**A**) i: Sample is strained until failure and exhibits large plastic before failure. ii: After exposure to an AMF for 30 s, the sample shows shape recovery of the plastically deformed regions without joining of the two halves. iii: Further exposure to the AMF for 20 minutes resulted in healing at the damaged interface. (**B**) PL spectra of Magpol shows the change in the I_E_ intensity (at 515 nm) before and after damage as well as after healing. (**C**) Mechanical properties after healing: Loads/extension curves of Magpol, until failure, healed and measured again until failure.

**Figure 6:**
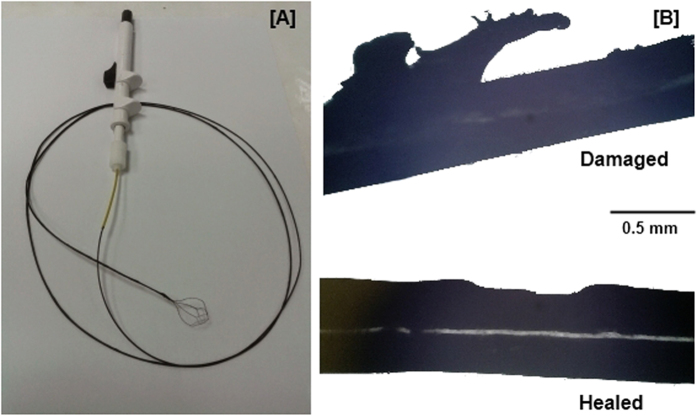
(**A**) NiTiNOL guidewire coated with 20 wt% Magpol. (**B**) Damaged portions of the coated wire were subjected to recovery in an AMF for 20 min, resulting in shape memory assisted self healing.

EVA has a hard/soft segment structure, the hard segment is elastic, the soft segment can significantly alter its stiffness when being heated above its melting temperature. Thus, EVA shows a dual-component mechanism of shape recovery^48^. The magnetic nanoparticles can act as net points which are required to store elastic energy, providing the driving force for shape recovery. This explains both the increase in storage modulus as well as greater shape recovery with increasing filler concentration (Fig. 2).

Strain sensing in Magpol depends on the sensitivity of the BBS aggregate to plastic deformation. During plastic deformation, there is macromolecular chain slippage and reorganization, which promotes break-up of non-covalent interactions among chromophore molecules and mixing of these molecules within the polymer matrix. The magnitude of this effect depends on the degree of crystallinity and mobility of the polymer chains.

The DSC analysis shows that there is a slight decrease in the crystallinity of Magpol during plastic deformation. This can provide greater volume for disaggregation of the chromophore molecules into the matrix. The almost linear decrease in the I_E_/I_M_ ratio (upto 150%), followed by a more gradual decrease, can be explained by the semicrystalline nature of the EVA matrix. The initial deformation of semi-crystalline polymers is by a scheme of inter and intra-lamellar shear of the lamellae. Firstly, deformation is (visco-)elastic and caused by straining inter-lamellar amorphous domains. At yield, two lamellar deformation mechanisms occur. The first mechanism is chain slip through the lamellae, called fine slip. This is responsible for the linear strain sensing behaviour of Magpol. After the second process of coarse slip of lamellae occurs, intra-crystalline shear results in fragmentation of lamellae, which does not affect the I_E_/I_M_ ratio but decreases the crystallinity of Magpol (DSC results in supplementary information)^49^.

The change in the shape memory behaviour with increasing filler loading is due to a decrease in crystallinity with increasing filler addition. The greater amorphous content results in more disaggregation of chromophores and higher shape recovery on applying the AMF. Thus, the chromophore based strain sensor acts as an early warning system of plastic deformation before failure.

The second step of healing in Magpol can be explained by the reptation theory, which states that, at temperatures above the glass transition (Tg), polymer chains will diffuse across an interface and entangle with each other to produce a bond. Healing between polymer chains at the interface is achieved by the following steps: surface rearrangement, surface approach, wetting, diffusion and randomization^50^. The process is temperature dependent and increasingly efficient at higher temperatures. Magnetic particle heating is ideally suited to provide this temperature increase remotely and wirelessly.

The mechanochromic response of Magpol is also recovered during the first step of shape recovery. This is a novel finding which enables us to reuse Magpol over several cycles. The decrease in intensity of the I_E_ signal compared to that of the undeformed sample could be due to the healing process in which chain rearrangements and entanglement disrupt chromophore aggregates on a small scale. This effect can be overcome by annealing the films to regain the aggregate structure. Our previous work shows that Magpol can undergo healing over more than five cycles without deterioration of its mechanical properties^45^. Enabling repeated strain sensing along with recovery of mechanical properties is important to prolong the service life and reliability of many components in practical applications.

**Figure 7:**
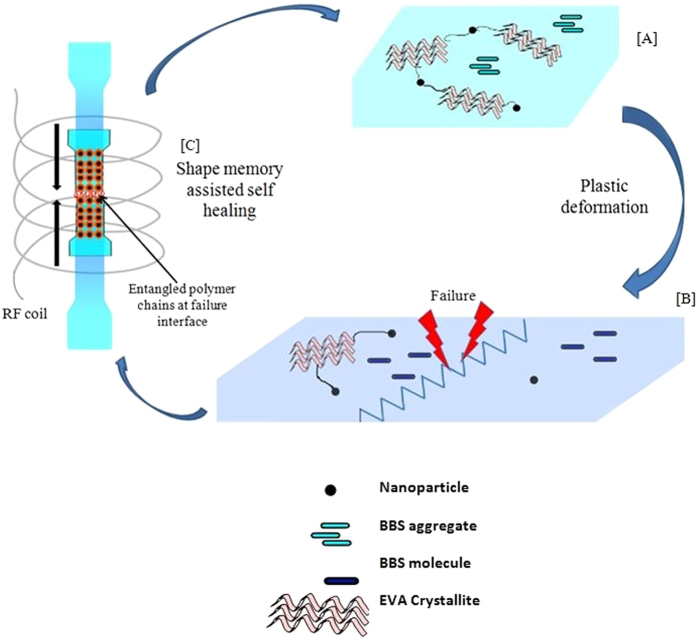
Model of recoverable strain sensing and self healing in Magpol. (**A**) Original: Sample shows high crystallinity, BBS molecules aggregated and nanoparticles form links between the polymer chains. (**B**) After plastic deformation and failure: a decrease in crystallinity, single BBS molecules formed due to disaggregation (resulting in a colour change). (**C**) Recovery: shape recovery causes recovery of the original dimensions. Polymer chain entanglement at the failure interface results in self healing BBS aggregates also re-form resulting in recovery of the original colour. Further cycles of strain and recovery follow the same pattern described above.

Figure 7 illustrates our model of the processes occuring during strain and recovery of Magpol. During plastic deformation, BBS chrompophore aggregates present in the amorphous part of the EVA break up into single molecules with a corresponding change in the emission spectrum. This is also accompanied by alignment of polymer chains and crystallites along the strain direction. The nanoparticles may also act as crosslinkers in the matrix. On triggering heating by exposure to the magnetic field, Magpol recovers its original shape, though some of the crystallites may still retain their orientation. Also, a few molecules of the chromophore may remain in the monomeric form. Subsequent cycles of strain and recovery follow the same path as the first cycle.

## Conclusions

We have developed a novel multicycle damage sensing and remotely triggered healing magnet-polymer composite material (Magpol). The material is capable of sensing deformation induced damage and can also repair the damage by means of an external AMF. By selecting a Curie temperature tuned magnetic nanoparticle filler, the heat produced by the particles in the AMF can be used to trigger a shape memory process followed by polymer chain entanglement. These two processes result in healing of the polymer matrix. The shape memory properties of EVA were exploited to trigger both recoverable strain sensing as well as self healing. The magnetic filler content influences the extent of shape recovery as well as healing efficiency. Interestingly, multiple cycles of failure and recovery did not adversely affect the sensing properties of the BBS chromophore. Healing, along with restoration of the mechanical properties of Magpol was achieved within 10 min. of exposure to the AMF. Thus, Magpol exhibits promise as a bioinspired multifunctional material.

## Experimental Methods

### Materials

All materials were used as received without further purification. manganese (II) chloride tetrahydrate 99% (MnCl_2_. 4H_2_O), zinc chloride, anhydrous (98+%) (ZnCl_2_), iron (III) chloride hexahydrate, ACS (FeCl_3_. 6H_2_O), sodium hydroxide (NaOH) were purchased from Alfa Aesar. 1,1,2,2 tetrachloroethane and tetrahydrofuran, anhydrous 99% was obtained from Sigma Aldrich. Polyethylene vinyl acetate of brand Cosmothene EVA KA-31 (28% VA content) and MilliQ water was used for all experiments.

### Nanoparticle synthesis

Nanoparticles were synthesized via the hydrothermal method. Manganese chloride (MnCl_2_)(80 mM), zinc chloride (ZnCl_2_)(20 mM) and iron chloride salts (FeCl_3_)(200 mM) were each dissolved separately in MilliQ water. NaOH (~5 M) was added to the iron chloride solution until a pH value of 8 was reached. The resulting precipitate was centrifuged and washed thrice with MilliQ water before being added to the Mn and Zn salt solutions. The salt solutions were then added together in a beaker equipped with a mechanical stirrer and vigorously stirred while adding sodium hydroxide drop wise, until the reaction mixture reached a pH of 12. The resulting slurry was decanted into a pressure vessel and placed in an oven at 190 °C for 4 h. These particles were then washed several times with DI water and ethanol and vacuum dried for further characterization.

### EVA film synthesis

EVA-magnetic particle composites were prepared by solution casting. The polymer pellets were dissolved in 1,1,2,2 tetrachloroethane (TCE) at ~120 °C. The appropriate amount of nanoparticles was then added to the solution which was then immediately cast into petri dishes in an oven at 120 °C. The TCE was allowed to evaporate for 4 h and the films were slowly cooled to room temperature. The resulting films were then peeled off and placed in a vacuum oven at room temperature for 24 h to completely remove the solvent. Further annealing was carried out in an oven at 60 °C for 1 h. The films obtained through this method were ~150 μm thick. Composite films containing 12 wt%, 16 wt.% and 20 wt% of nanoparticles and different wt% of 4,4′-Bis(2-benzoxazolyl)stilbene (BBS) were cast for further study.

### Characterization

The nanoparticle filler was characterized by X-ray diffraction (XRD), vibrating sample magnetometer (VSM) and transmission electron microscopy (TEM) (supplementary information). The Magpol composite was characterized by differential scanning calorimetry (DSC) (supplementary information), dynamic mechanical analysis (DMA) and tensile testing.

### DMA (shape memory)

Dynamic mechanical analysis (DMA) was used to determine the glass transition temperature (Tg). Tg can be used to examine the effect of the filler on the shape memory performance. All samples were studied using a TA Instruments Q800 Dynamic Mechanical Analyzer with a film tension accessory. The test was run in Multi-frequency Strain mode to obtain Tg at a maximum frequency of 0.1 Hz. To monitor shape memory strain, the instrument was run in Controlled Force mode. Two cycles of heating and cooling were carried out; stresses and resulting deformation strains were measured.

### Strain sensing

To evaluate the strain sensing properties of Magpol, the film was cut into dogbone shaped samples and strained under a crosshead speed of 25 mm/min in a tensile tester (Instron 5567). A draw ratio of ~3 was used in each case. The fluorescence of the film was measured by a spectrofluorophotometer (Shimadzu RF-5301) at an excitation wavelength of 377 nm; the measurements were carried out both before and immediately after film straining.

### Self healing

Magpol was fractured by straining the samples until failure, using an Instron Mechanical Tester 5567 with a 500N load cell and a crosshead speed of 50 mm/min. Healing was achieved by holding the torn edges of the sample together between two glass slides. The sample was then placed within a water-cooled 5-loop copper induction coil energized by an AC generator (Inductelec, UK) with an operating frequency of 475 kHz and healing time of 10 min.

## Additional Information

**How to cite this article**: Ahmed, A. S. and Ramanujan, R. V. Magnetic Field Triggered Multicycle Damage Sensing and Self Healing. *Sci. Rep.* **5**, 13773; doi: 10.1038/srep13773 (2015).

## Supplementary Material

Supplementary Information

## Figures and Tables

**Table 1:** Shape fixity and recovery values of 12 wt%, 16 wt% and 20 wt% Magpol over 3 cycles.

Wt. Loading %	Shape Fixity (%)	Shape Recovery (%)
20	63.8	100
16	69.7	97.1
12	59.5	96.7
