# Supplementary material for: Magnetic Field Triggered Multicycle Damage Sensing and Self Healing
Source: Sci Rep. 2015 Sep 8;5:13773. doi: 10.1038/srep13773 (PMC4562241; doi:10.1038/srep13773)
Supplement: Supplementary Information [file srep13773-s1.doc]

Supplementary information


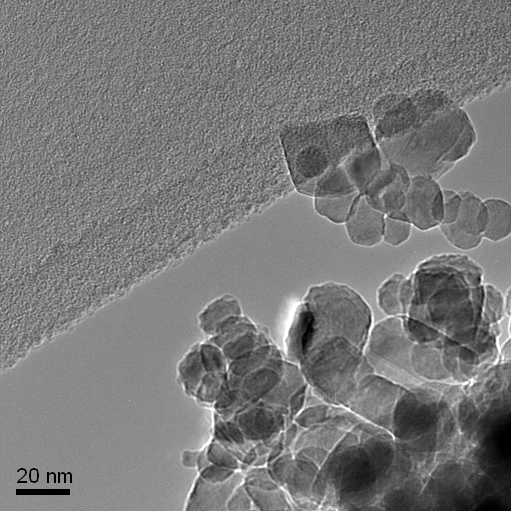


**Figure S1: Bright field TEM micrograph of Mn-Zn Ferrite**


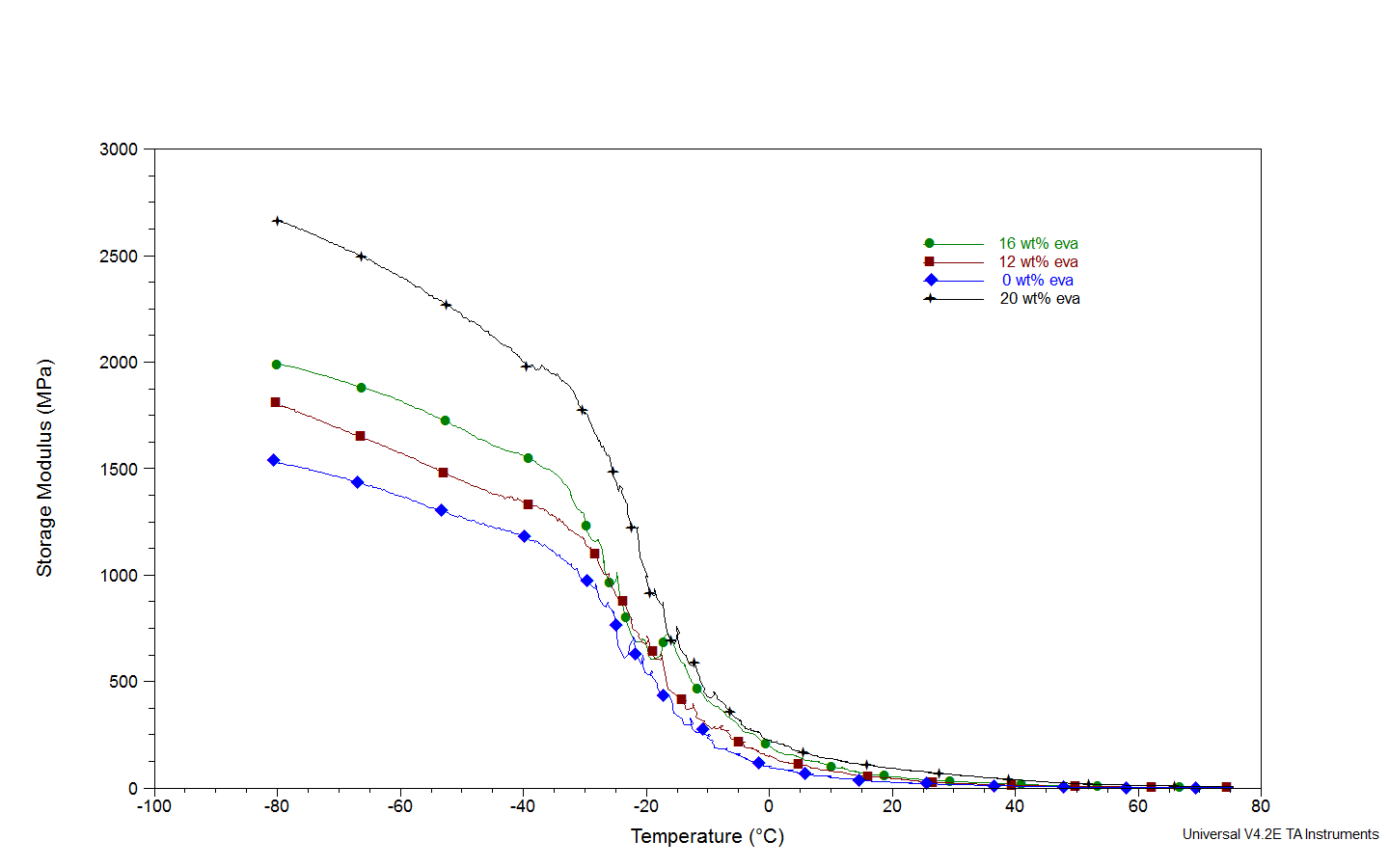


**Figure S2: Change in storage modulus for varying filler loading in the range of 0 to 20 wt% with temperature**


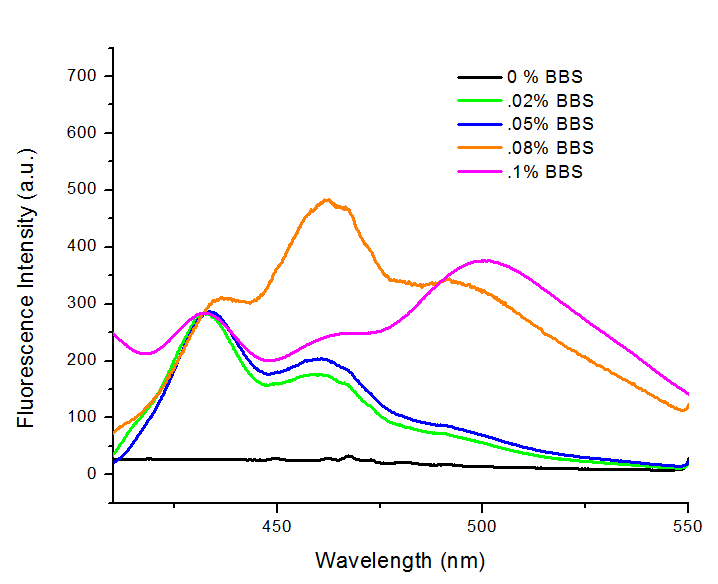


**Figure S3: PL spectra of Magpol with varying BBS concentration**


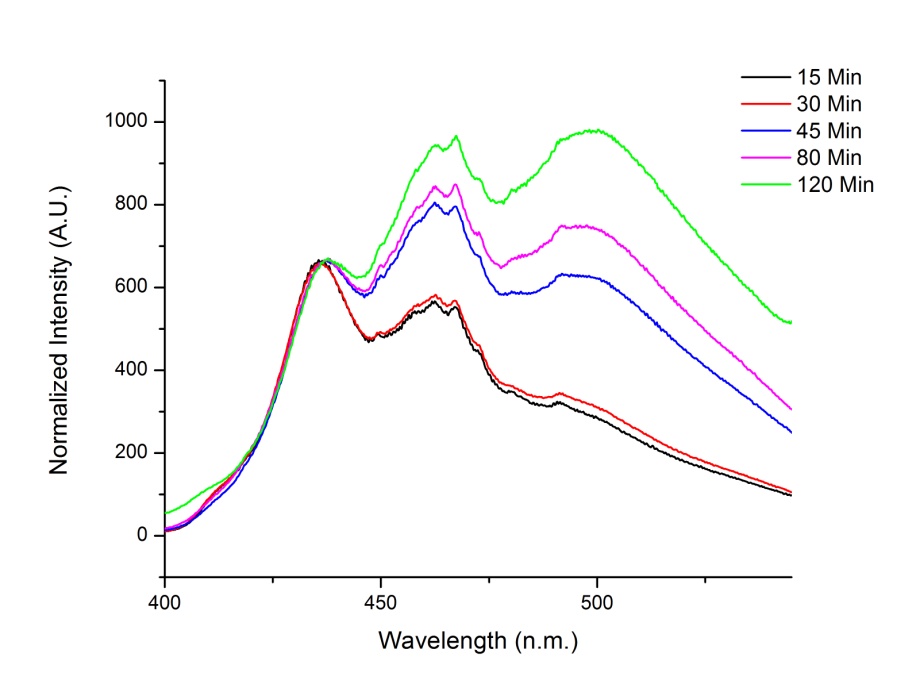


**Figure S4: Change in Magpol PL spectra with annealing time**


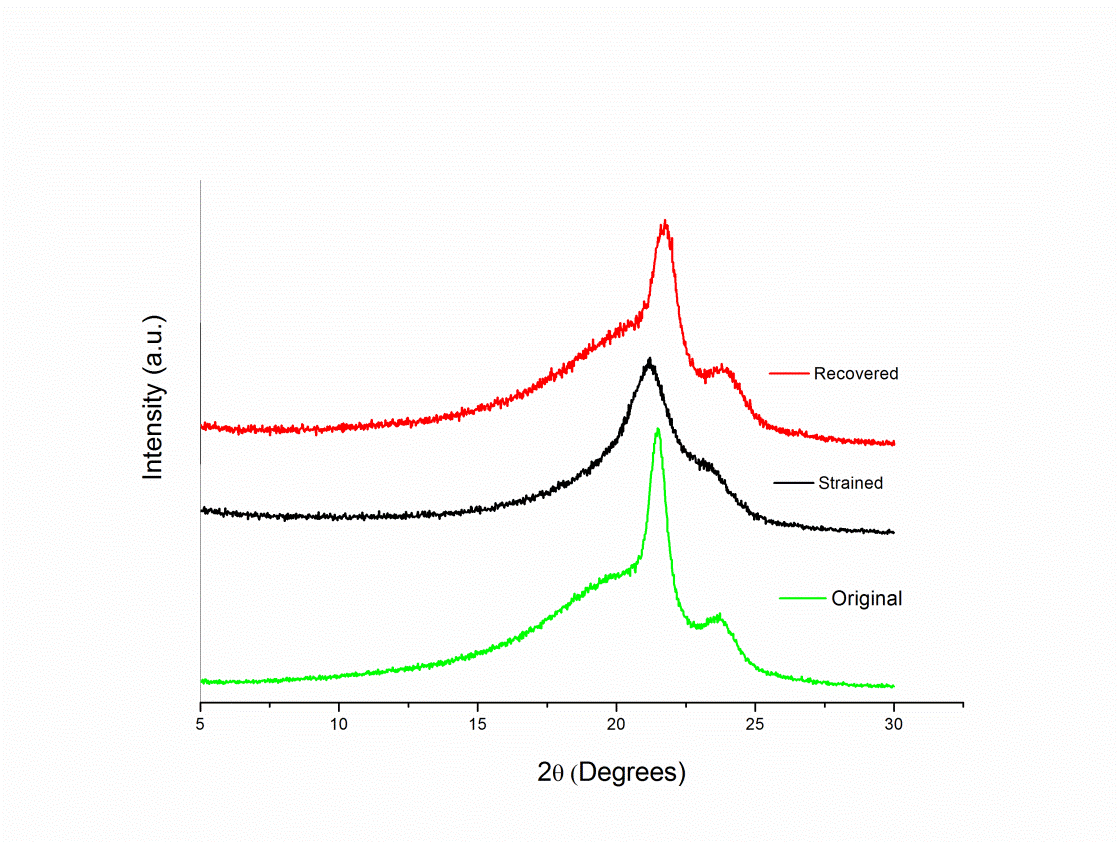


Figure S5: XRD of Magpol before strain damage, after damage and after recovery showing decrease in crystallinity

**Table S1**: Percent crystallinity of Magpol with different filler loadings before and after damage recovery

| Loading wt% | Sample condition | % Crystallinity |
| --- | --- | --- |
| 12 | Original | 10.17 |
|  | Stretched | 9.26 |
|  | Recovered | 6.97 |
| 16 | Original | 12.14 |
|  | Stretched | 7.87 |
|  | Recovered | 6.49 |
| 20 | Original | 8.5 |
|  | Stretched | 8.13 |
|  | Recovered | 3.88 |


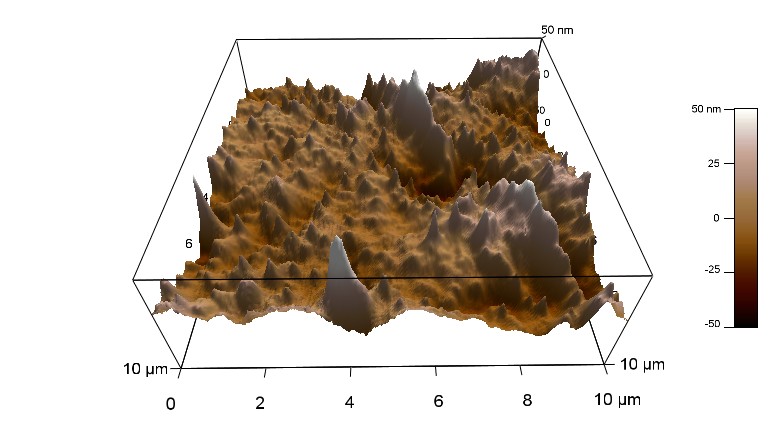

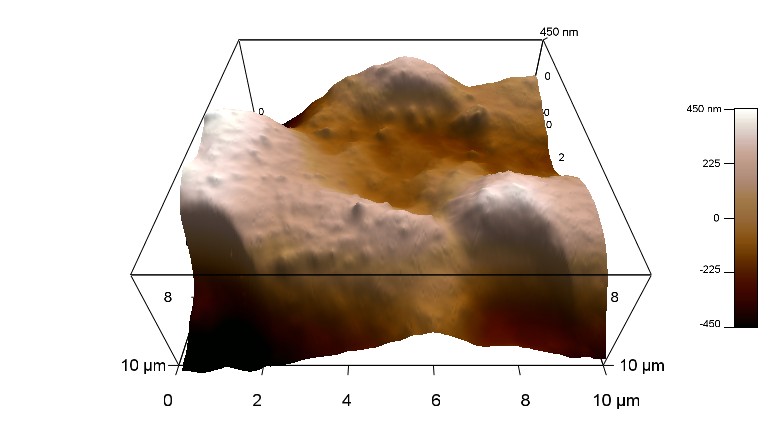

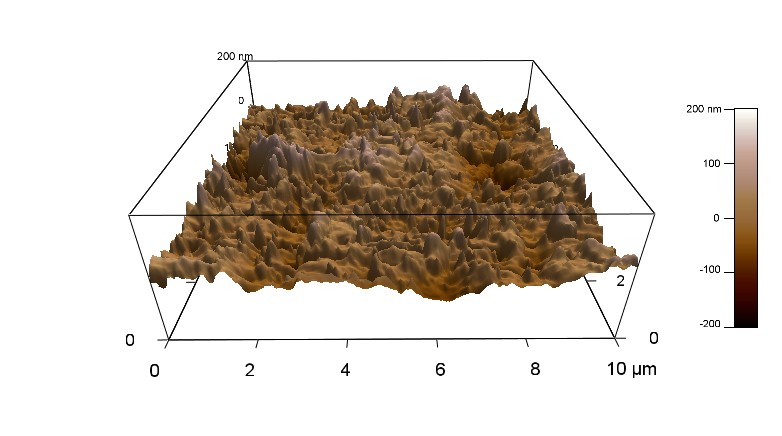


(a)

(b)

(c)

**Figure S6: AFM images showing BBS chromophore aggregates a) before damage b) after damage and c) after recovery**

**
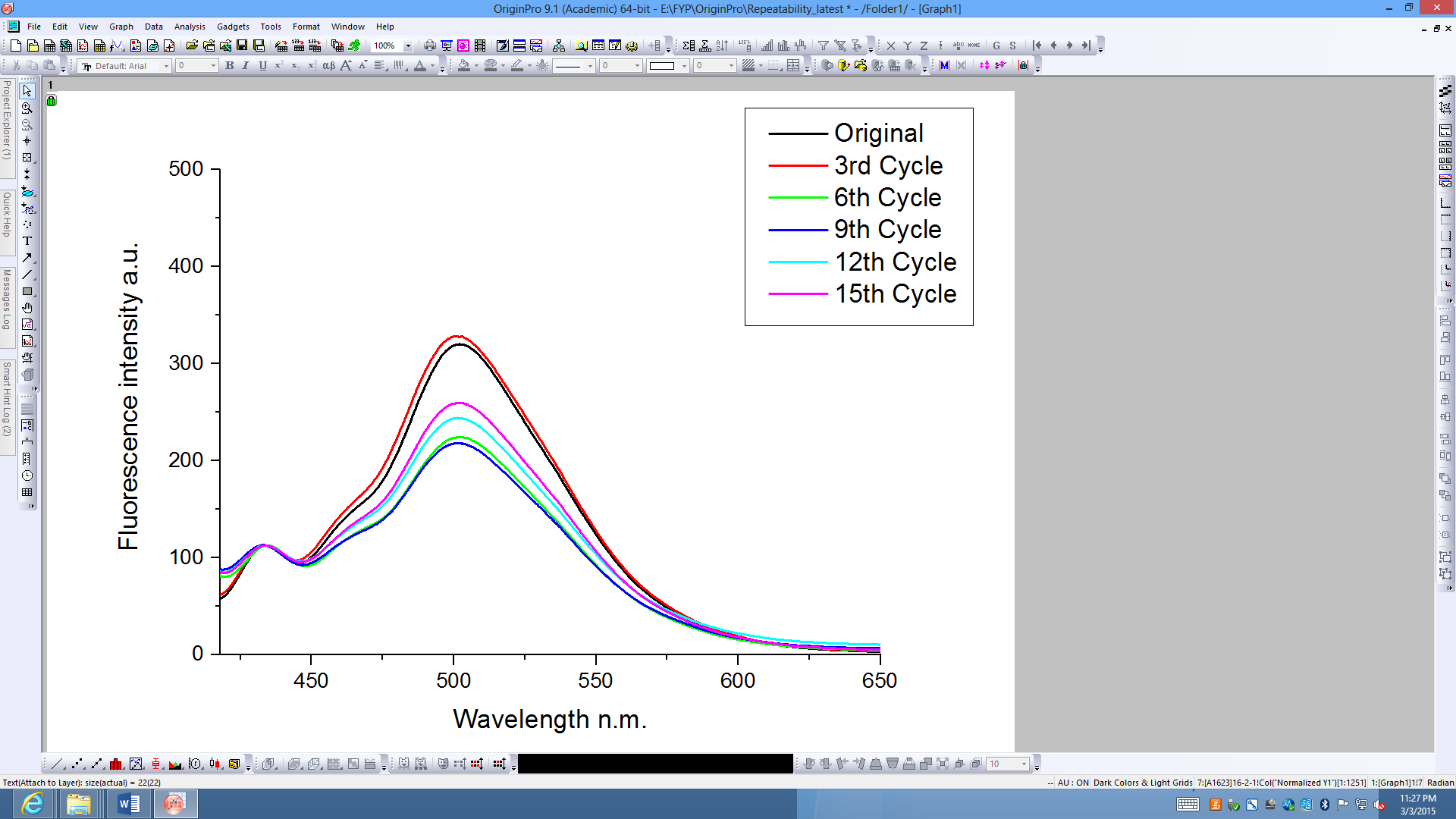
**

**Figure S7: PL spectra of Magpol after recovery through 15 cycles.**


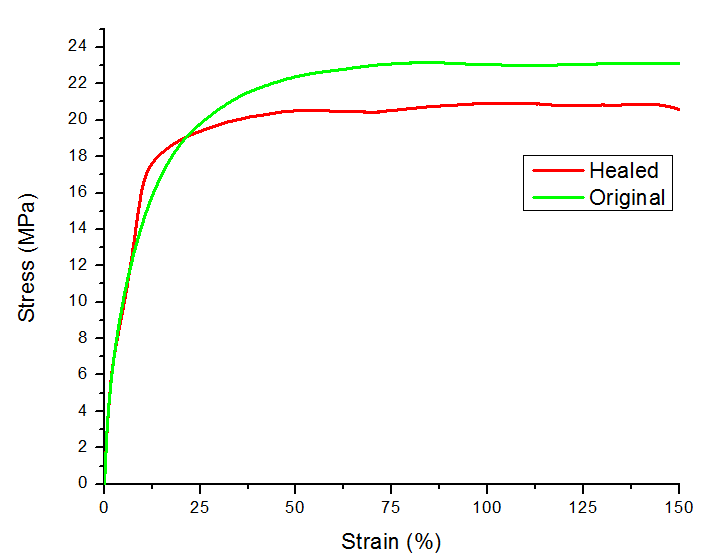


**Figure S8: Mechanical properties of Magpol guidewire before damage and after recovery**
